# Supplementary material for: Development of Fractalkine-Targeted Nanofibers that Localize to Sites of Arterial Injury
Source: Nanomaterials (Basel). 2020 Feb 28;10(3):420. doi: 10.3390/nano10030420 (PMC7152859; doi:10.3390/nano10030420)
Supplement: Supplementary file 1 [file nanomaterials-10-00420-s001.pdf]

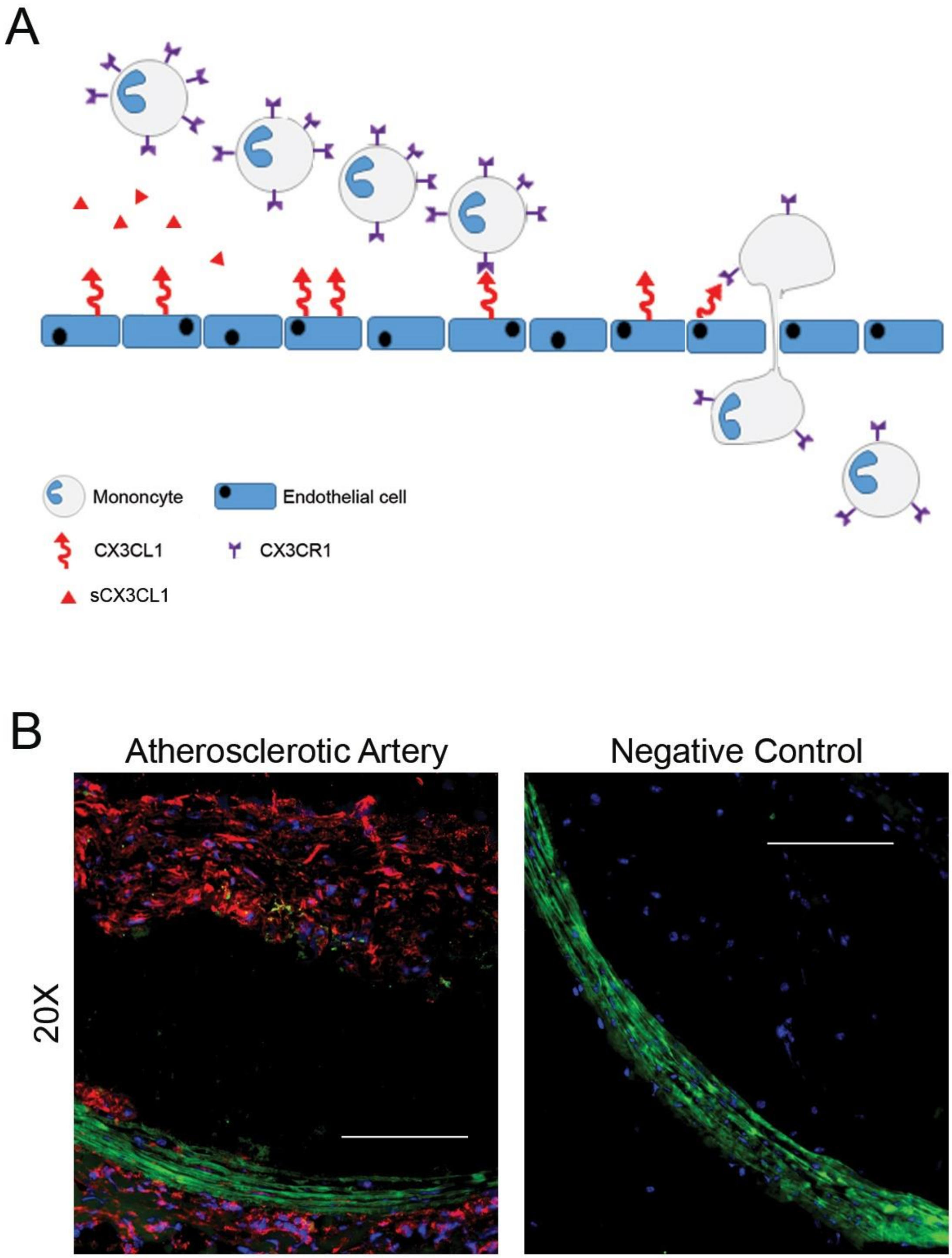

Figure S1. (**A**) Schematic of fractalkine (CX3CL1) and its associated receptor (CX3CR1) on monocytes. Monocytes attracted to the area by soluble CX3CL1 (sCX3CL1), attach to activated endothelial cells through fractalkine, and undergo diapedesis to enter the subendothelial space. (**B**) Positive staining for fractalkine in atherosclerotic artery with no staining seen in negative control artery (secondary antibody only). Fluorescent microscopy of atherosclerotic artery in LDLR-/- mice fed a high-fat diet for 16 weeks, stained for CX3CR1. Green = autofluorescence of arterial lamina, blue = DAPI nuclear stain, and red = Alexa546/CX3CR1 antibody. 20X magnification, n=3/treatment group. Scale bar = 100μm.

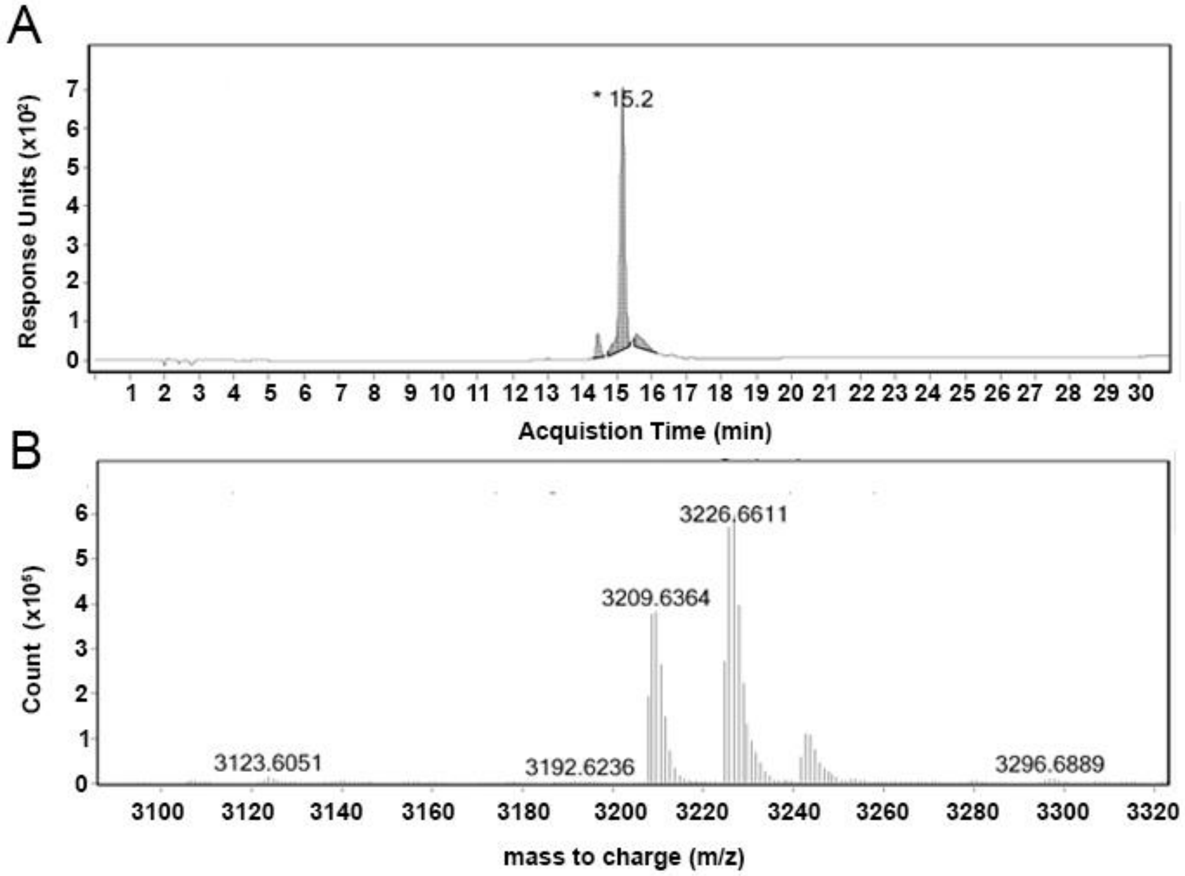

Figure S2. LCMS characterization of the fractalkine-targeted PA nanofiber. (**A**) HPLC trace of the fractalkine-targeted PA shows 90% purity. (**B**) Deconvoluted electrospray ionization (ESI) mass spectrometry (MS) of targeted nanofiber shows peaks at the expected mass (3209 and 3226 (NH3 adduct) m/z) for fractalkine-targeted PA.

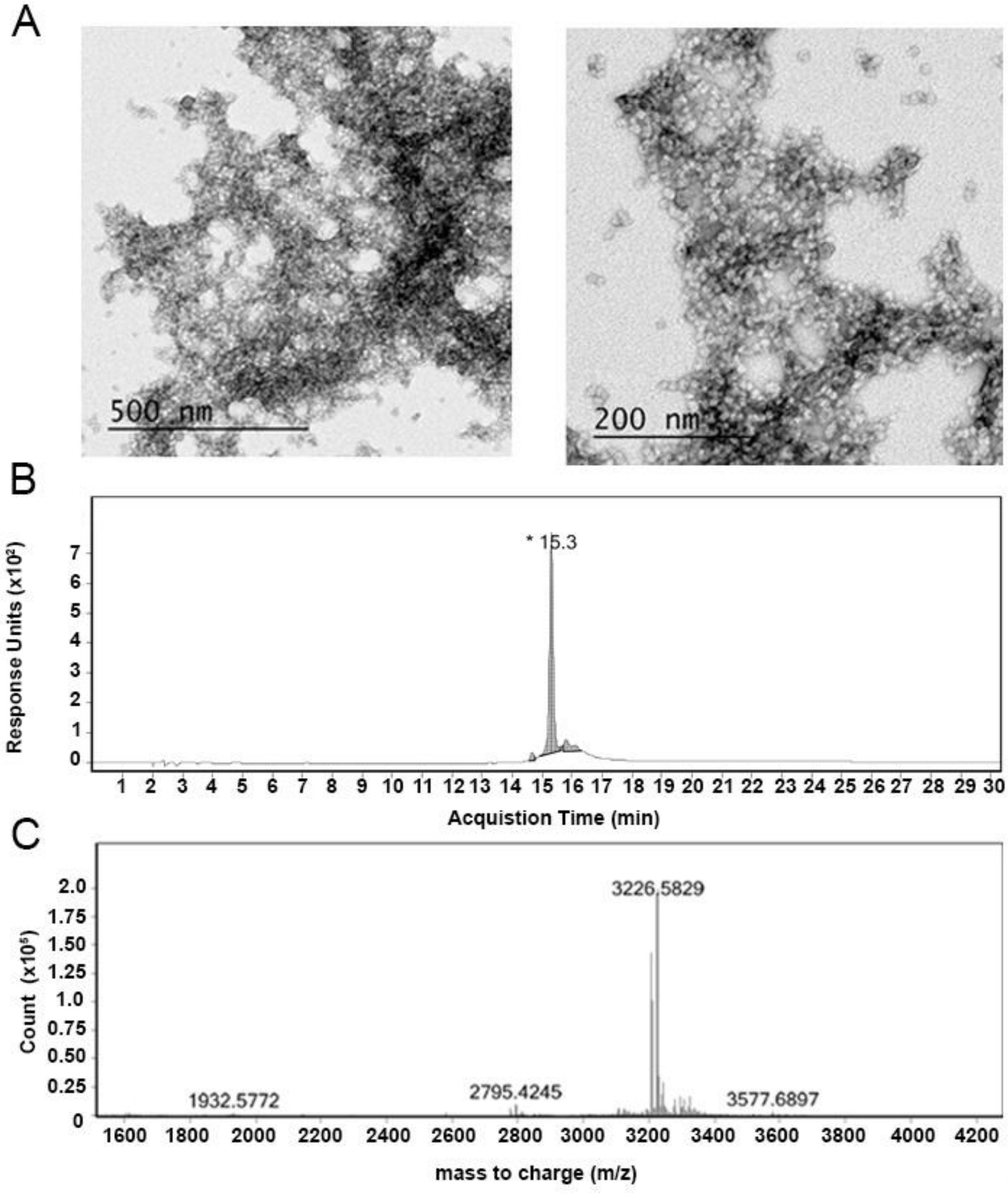

Figure S3. Characterization of the scrambled PA nanofiber. (**A**) Conventional transmission electron microscopy (TEM) images of scrambled nanofibers. (**B**) HPLC trace of the scrambled PA shows 92% purity. (**C**) Deconvoluted electrospray ionization (ESI) mass spectrometry (MS) of scrambled nanofiber shows peaks at the expected mass (3226.58) for the scrambled PA.

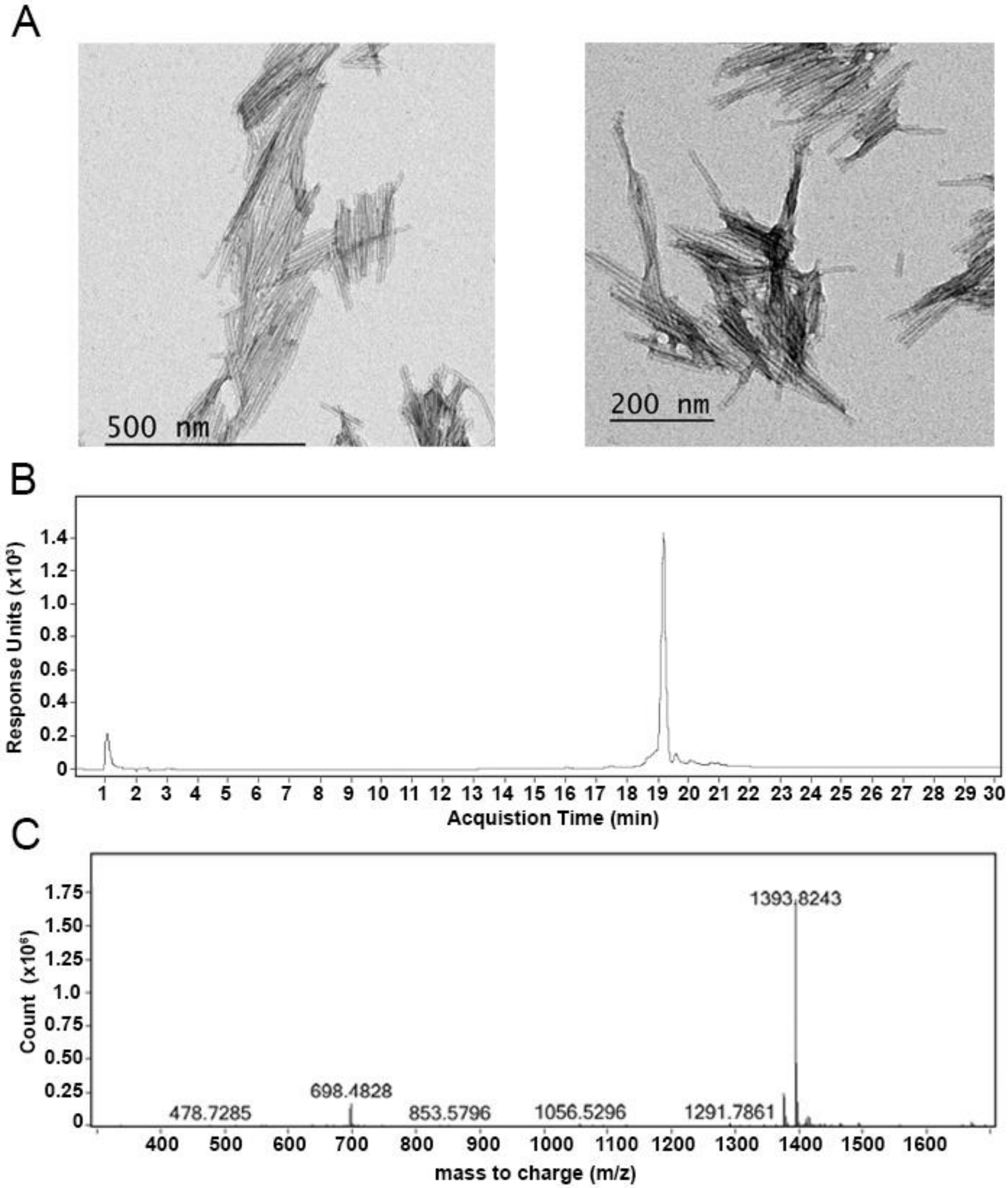

Figure S4. Characterization of the backbone PA nanofiber. (**A**) Conventional transmission electron microscopy (TEM) images of the backbone PA nanofiber. (**B**) HPLC trace of the backbone PA shows greater than 92% purity. (**C**) Deconvoluted electrospray ionization (ESI) mass spectrometry (MS) of backbone nanofiber shows peaks at the expected mass (1393 m/z) for the backbone PA.

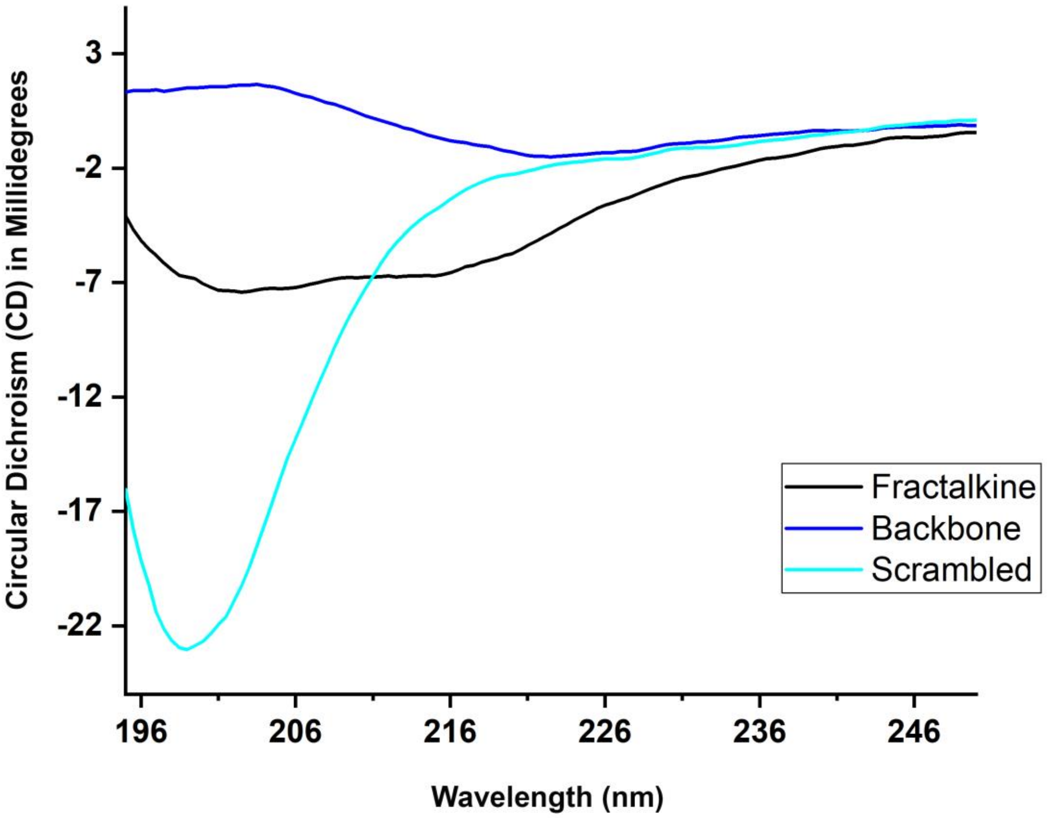

Figure S5. Circular dichroism spectroscopy demonstrates that the backbone PA nanofiber exhibits beta-sheet formation, the fractalkine targeting PA nanofiber exhibits α-helical characteristics, and the scrambled PA nanofiber exhibits random coils.
